# Liver organoids: a promising three-dimensional model for insights and innovations in tumor progression and precision medicine of liver cancer

**DOI:** 10.3389/fimmu.2023.1180184

**Published:** 2023-06-02

**Authors:** Yukun Chen, Yujun Liu, Shimin Chen, Long Zhang, Jiawei Rao, Xinjun Lu, Yi Ma

**Affiliations:** ^1^ Organ Transplant Center, The First Affiliated Hospital, Sun Yat-sen University, Guangzhou, China; ^2^ Zhongshan School of Medicine, Sun Yat-sen University, Guangzhou, China; ^3^ Guangdong Provincial Key Laboratory of Organ Donation and Transplant Immunology, The First Affiliated Hospital, Sun Yat-sen University, Guangzhou, China; ^4^ Guangdong Provincial International Cooperation Base of Science and Technology (Organ Transplantation), The First Affiliated Hospital, Sun Yat-sen University, Guangzhou, China; ^5^ Department of Biliary-Pancreatic Surgery, Sun Yat-sen Memorial Hospital, Sun Yat-sen University, Guangzhou, China

**Keywords:** organoid, liver cancer, tumor microenvironment, personalized treatment, precision medicine

## Abstract

Primary liver cancer (PLC) is one type of cancer with high incidence rate and high mortality rate in the worldwide. Systemic therapy is the major treatment for PLC, including surgical resection, immunotherapy and targeted therapy. However, mainly due to the heterogeneity of tumors, responses to the above drug therapy differ from person to person, indicating the urgent needs for personalized treatment for PLC. Organoids are 3D models derived from adult liver tissues or pluripotent stem cells. Based on the ability to recapitulate the genetic and functional features of *in vivo* tissues, organoids have assisted biomedical research to make tremendous progress in understanding disease origin, progression and treatment strategies since their invention and application. In liver cancer research, liver organoids contribute greatly to reflecting the heterogeneity of liver cancer and restoring tumor microenvironment (TME) by co-organizing tumor vasculature and stromal components *in vitro*. Therefore, they provide a promising platform for further investigation into the biology of liver cancer, drug screening and precision medicine for PLC. In this review, we discuss the recent advances of liver organoids in liver cancer, in terms of generation methods, application in precision medicine and TME modeling.

## Introduction

Primary Liver cancer (PLC) is the third leading cause of cancer death worldwide, only behind lung cancer and colorectal cancer (CRC). In 2020, approximately 906,000 new cases and 830,000 deaths were recorded (1). Hepatocellular carcinoma (HCC) makes up 80% of PLC, followed by intrahepatic cholangiocarcinoma (iCCA, 10%) and other rare types (2). The main risk factors causing PLC are infection with hepatitis B virus (HBV), or hepatitis C virus (HCV), alcohol abuse, non-alcoholic fatty liver disease, dietary exposure to aflatoxin B etc. (3–5). Due to the strong compensation ability and large functional reserve of liver, nearly half of patients are in advanced stage of HCC once diagnosed and may lose the chance to receive conventional therapy, including radical resection and ablation (6). Although advanced-stage HCC can benefit from systemic therapy including targeted therapy and immunotherapy, choices are quite limited. Sorafenib, a multikinase inhibitor, remained the solely first-line option for nearly a decade until IMbrave 150 trial demonstrated an improvement in overall survival with atezolizumab plus bevacizumab (19.2 months versus 13.4 months) (7–9). However, there is still insufficiency of global understanding of immune checkpoint inhibitor resistance (10). Considering that PLCs are tumors with high intra-tumor heterogeneity and complex TME, the key to implement precision medicine in PLC treatment is to utilize reasonable pre-clinical models, which could recapitulate the molecular and structural features of patients’ tumor. Hence, high throughput screening (HTS), drug screening, and novel biomarkers exploration could be achievable and extensively promoted.

Another question emerging in current research is that tumor cells are not alone. They communicate with other cells in the niche, relying on cytokines and stromal components in the environment for survival and invasion (11). The conventional 2D cell model grows as a monolayer with a completely distinct cell-cell interaction pattern and cell-stromal relationship due to loss of polarity, altered gene expression profiles, disturbed signaling pathways, histological architecture etc. (12, 13) Thus, 2D models can’t maintain the pristine niche of primary tumors (14, 15). By contrast, 3D patient-derived organoids (PDOs) could provide a reasonable answer to the two questions above. In this review, we summarize different methods to generate liver organoids and the applications of PDOs in precision medicine of liver and TME research.

## Generation methods of liver organoids

The term “organoid” is clearly defined as “a 3D structure derived from (pluripotent) stem cells, progenitor, and/or differentiated cells that self-organize through cell-cell and cell-matrix interactions to recapitulate aspects of the native tissue architecture and function *in vitro*” *(* 16). According to different origins, there are generally two major kinds of liver organoids, liver tissue-derived organoids and pluripotent stem cell−derived organoids.

### Liver tissue-derived liver organoids

Early in 2015, Huch et al. established the first human liver organoids from adult bile duct-derived bipotent progenitor cells, which could expand and differentiate into functional hepatocytes with stable genetic and chromosomal features (17). However, compared with these intrahepatic cholangiocyte derived organoids (iCD) which have bipotent differentiation capacity, extrahepatic cholangiocyte derived organoids (eCD) can only differentiate towards a cholangiocyte fate (18, 19). The region-specific differentiation potential of these two types of organoids indicates that generation of liver organoids needs tissue-specific source of cells. Besides, fetal hepatocytes share closer relationship with three-dimensional liver bud than adult cells (20) and can also be utilized for long-term expansion into organoids (21). What’s more, tumor organoids (tumoroids) have been recently developed and extensively applied into basic research (22). Broutier et al. are the first to generate human primary liver cancer–derived organoids from surgical resected tumor tissues (23). Needle biopsy is another method with equivalent practical value as well (24). Recently, Narayan et al. established fibrolamellar carcinoma organoids from resection or biopsy derived primary and metastatic tissues for investigation of this rare and lethal kind of HCC frequently occurring in adolescents (25). In addition, liver tumoroids can also be obtained from iPSC-derived liver organoids by overexpression of a oncogene, c-myc (26).

Aside from human cell-derived organoids, murine liver organoids have been studied for a long time. Isolated Lgr5+ cells from damaged mouse liver biliary ducts carry the hallmarks of bipotent liver progenitors. Therefore, these cells can be expanded into organoids by coculturing in the medium based on Rspo1, which is a key activator of Wnt signaling, a crucial pathway for cell self-renewal and development (27). Recently, murine hepatocyte-derived organoids were successfully constructed to mimic the regenerative responses during liver injury (21). Moreover, Cao et al. reported the establishment of primary mouse liver tumor cell-derived organoids for tumorigenesis research and drug screening (28).

**Figure 1:**
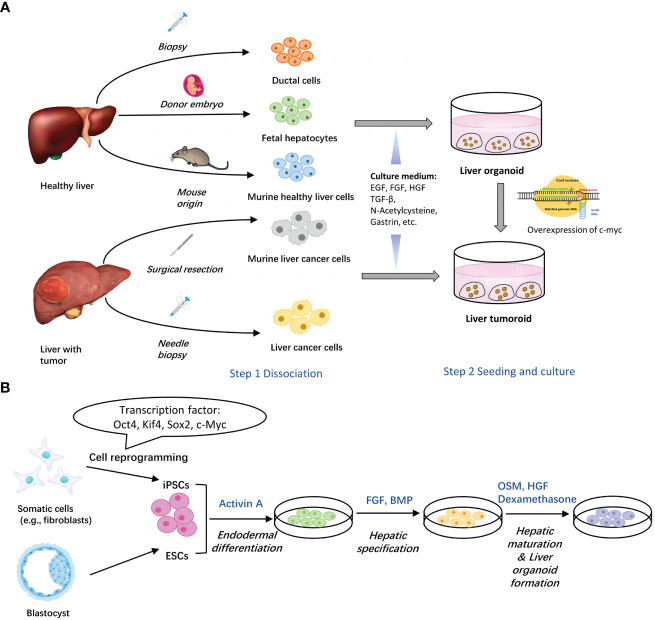
Two main methods for generation of liver organoids. **(A)** Establishment of liver organoids from adult liver tissues. Healthy liver cells or liver cancer cells can be obtained *via* surgical resection and biopsy. Upon seeded into Matrigel with different culture components, cells assemble themselves into organoids. Liver tumoroids can be induced from liver organoids by overexpression of c-myc. **(B)** Establishment of liver organoids from stem cells. Both of ESCs and iPSCs can be utilized to generate liver organoids through three major steps, endodermal differentiation, hepatic specification and hepatic maturation & liver organoid formation. Each step contains several key factors. EGF, epidermal growth factor; FGF, fibroblast growth factor; HGF, hepatocyte growth factor; TGFβ, transforming growth factor beta; BMP, bone morphogenetic protein; FBS, fetal bovine serum.

To sum up, generation of organoids from tissues usually involves two basic steps, isolation of single liver cells and seeding of cells into culture medium (15). (**Figure 1A**) Commonly added elements include AdDMEM/F12, N2/B27 supplement, N-Acetylcysteine, gastrin, multiple growth factors (epidermal growth factor, fibroblast growth factor, hepatocyte growth factor), RSPO1, A83-01, FSK etc. (29, 30) (**Table 1**).

**Table 1:** Summary of culture medium components for generation of liver tissue-derived liver organoids.

		Function of supplemented components	liver organoids				liver cancer organoid		
References			Huch et al. (17)	Hu et al. (21)	Hu et al. (21)	Lam et al. (30)	Broutier et al. (23)	Cao et al. (28)	Wang et al. (29)
Source cells or tissues			Liver ductal cells	Fetal hepatocytes	primary mouse hepatocytes	Nontumoral human liver tissues at the resection margin of surgical HCC	Liver tumor specimens through resection	Primary mouse liver cancer tissues	Hepatobiliary tumor specimens through resection
Digestion			collagenase-accutase	Type IV collagenase	Type IV collagenase	collagenase type II	collagenase-accutase	0.5 mg/ml Collagenase type XI; 0.2 mg/ml Dispase; 1% FBS in DMEM	collagenase D ; 0.1 mg mL−1 DNase I ; 2 × 10−6 m Y27632; 100 µg mL−1 Primocin
Basement membrane simulation			Matrigel or reduced growth factor BME 2	Matrigel	Matrigel	Matrigel	BME2	Matrigel	Matrigel
Culture media	AdDMEM/F12	Provision of basal nutrition	√	√	√		√	√	√
	AIM-V	Provision of basal nutrition without serum				√			
	Penicillin-Streptomycin	Antibiotics to prevent of microorganism contamination		√	√	√(0.35 U/ml )	√(1%)		
	L-Glutamine	Essential amino acid for cell growth				√			
	Glutamax	Substitution of L-Glutamine		√	√		√(1%)		√(1%)
	HEPES	Buffering agents to stablize the culture medium		√	√		√(10mM)		√(1mM)
	1% N2	Suppresion of cell differentiation and promotion of cell groeth	√			√	√	√	
	B27	Suppresion of cell differentiation and promotion of cell groeth	√(1%)	√(1%, minus vitamin A)	√(minus vitamin A)	√(1%)	√(2%, minus vitamin A)	√(2%)	√(2%, minus Vitamin A)
	1× MEM NEAA	Promotion of cell viability				√			
	1× ITS	Insulin: Promotion of energy metabolism; Transferrin: Carrier of Iron and lowering of oxygen radicals; Selenium: Anti-oxidant				√			
	3 μm CHIR99021	GSK3β inhibitor to activate Wnt pathway		√	√				
	100 µg mL−1 primocin	Antibiotics to prevent of microorganism contamination							√
	N-Acetylcysteine	Regulate cell proliferation, differentiation and apoptosis	√(1.25mM)	√(1.25mM)	√(1.25mM)	√(1mM)	√(1.25mM)	√(1.25mM)	√(1.25mM)
	10 nM gastrin	Promotion of expention of stomach cells	√	√	√	√	√	√	
	EGF	Ligands of receptor protein tyrosine kinase; promotion of expansion of epithelial cells	√(50ng/ml)	√(50ng/ml)	√(50ng/ml)	√(100ng/ml)	√(50ng/ml)	√(50ng/ml)	√(50ng/ml)
	FGF-basic	Fibroblast growth factor; Ligands of receptor protein tyrosine kinase							√(1ng/ml)
	FGF7	Fibroblast growth factor; Ligands of receptor protein tyrosine kinase		√(100ng/ml)	√(50ng/ml)				
	FGF10	Fibroblast growth factor; Ligands of receptor protein tyrosine kinase	√(100ng/ml)	√(100ng/ml)	√(50ng/ml)	√(100ng/ml)	√(100ng/ml)	√(100ng/ml)	√(100ng/ml)
	HGF	Hepatic growth factor	√ (25ng/ml)	√(50ng/ml)	√ (25ng/ml)	√ (25ng/ml)	√ (25ng/ml)	√(50ng/ml)	√(25ng/ml)
	RSPO1 conditioned media	Wnt activator	√(10%)	√(15%)	√(15%)	√(10%)		√(10%)	√(10%)
	10mM Nicotinamide	Sirtuins inhibitor to promote of self-renewal of HCC stem cells and cell energy metabolism	√	√	√	√	√	√	√
	A8301	TGFβ inhibitor to promote cell expansion and inhibit apoptosis and differentiation	√(5uM)	√(2uM)	√(1uM)	√(3uM)	√(5uM)		√(5μM)
	10 μM Forskolin	Rho Inhibitor (cAMP activator)	√			√	√		√
	10 μM γ-27632	Rock inhibitor to inhibit apoptosis of stem cells	√	√	√		√		√
	Noggin	BMP-4 and BMP-7 inhibitor to inhibit the differentiation of stem cells	√ (25 ng/ml)			√ (25 ng/ml)		√(10%)	√(5%)
	30% Wnt Conditioned medium	Ligand of canonical Wnt/β-catenin pathway to promote cell expansion	√			√		√	√
	20ng/ml TGF-α	Regulators of cell proliferation and differentiation		√					
	10ng/ml Oncostatin M	Inhibitors of proliferation of a number of tumor cell lines		√					
	Dexamethasone	Induction of apoptosis of hepatic cancer cells		√(1uM)			√(3uM)		

BME2, Basement Membrane Extract, Type 2; NEAA, Non-Essential Amino Acids; ITS, Insulin-Transferrin-Selenium; EGF, epidermal growth factor; FGF, fibroblast growth factor; HGF, hepatocyte growth factor; TGFα, transforming growth factor alpha; BMP, bone morphogenetic protein.

√ indicates that the culture medium contains the relevant components.

### Pluripotent stem cell-derived liver organoids

Pluripotent stem cells used to generate organoids include embryonic stem cells (ESCs) and induced pluripotent stem cells (iPSCs). ESCs are obtained from the inner cell mass of blastocyst and can be conditioned into hepatocytes *in vitro*. However, there exists multiple concerns about the use of ESCs, including ethical problems, risk of teratoma formation and tumorigenicity (31), which constrains its application in the clinical setting. iPSCs, however, have been widely investigated in research as an alternative to ESCs, due to the lack of ethical controversy and its compatibility with human body when used as autologous grafts.

Early in 2011, Amimoto et al. developed the hollow/organoid culture method to induce the hepatic differentiation of murine ESCs and iPSCs, and obtained cylindrical organoids with high cell density but poor hepatic functions and gene expression profiles (32). To improve, by using Nanopillar Plate, PSC-derived functional hepatocytes assembled into 3D spheroids with good prediction value for drug hepatotoxicity (33). Shortly afterwards, by coculturing iPSC-derived hepatic cells with mesenchymal stem cells and human umbilical vein endothelial cells, Takebe et al. successfully generated the first 3D vascularized and functional human liver buds which were able to mimic liver organogenesis and well recapitulate hepatic vascular network *in vivo (* 34). Nonetheless, these liver buds still retained some fetal characteristics with little proliferation capacity. Therefore, Mun et al. established functionally mature human hepatic organoids from ESCs and iPSCs with long term expansion ability and high fidelity to liver tissues (35). However, despite all these progressions, researchers found that gene expression profiles of PSC-derived liver organoids showed relatively strong distinction from primary human hepatocytes (34, 36). Therefore, researchers discovered some factors important for hepatic induction of iPSCs. For instance, expansion of nuclear receptor FXR would decrease the expression of intestine related genes so that to prevent unnecessary disturbance (37). Besides, mesoderm-derived paracrine signals are sufficient for hepatocyte maturation while organoid self-organization requires cell-to-cell surface contact (38). Hence, key factors to regulate hepatocyte differentiation should not be limited to just one element, but a group of genes or cells instead. Further research is in progress to dig them out (39–41) (**Figure 1B** and **Table 2**).

**Table 2:** Summary of culture medium components for generation of Pluripotent stem cell (PSC)−derived liver organoids.

References		Takebe et al. (39)	Mun et al. (35)	Yang et al. (41)	Ouchi et al. (42)	Guo et al. (40)
Steps	stem cells	iPSC	iPSC	iPSC	ESC/iPSC	iPSC
Endodermal differentiation	RPMI1640	√ (without insulin)	√ (without insulin)	√	√	√
	N2				√	
	B27	√	√		√	
	albumin fraction V					√
	PI-103			√		
	ITS					√
	100 ng/ml human activin A	√	√	√	√	√
	CHIR99021			√	√(3μM)	
Hepatic specification	RPMI1640	√	√			
	IMDM			√		
	B27	√	√	√		
	N2					
	FGF	√(10 ng/ml)	√(10 ng/ml)	√(10 ng/ml)		
	FGF4				√(500 ng/ml)	√(30 ng/ml)
	BMP4	√(20 ng/ml)	√(20 ng/ml)	√(20 ng/ml)	√(50 ng/ml)	
	BMP2					√(20 ng/ml)
	HGF			√(20 ng/ml)		√(20 ng/ml)
	KGF					√(20 ng/ml)
	FCS				√	
	5% hypoxia		√			
Hepatic maturation and liver organoid formation	Advanced DMEM/F12				√	√
	B27				√	√
	N2				√	√
	2μM retinoic acid				√	
	HCM without EGF	√	√		√	√
	EGM	√	√			
	FBS	√	√			
	HGF	√(10ng/ml)	√(10ng/ml)	√(20 ng/ml)	√(10ng/ml)	
	100 nM dexamethasone	√	√		√	√
	Oncostatin M	√(20 ng/ml)	√(20 ng/ml)	√(20 ng/ml)	√(20 ng/ml)	√(10ng/ml)
	1 mM/L glutamax					√
	1% NEAA					√
	0.1 mM β-mercaptoethanol					√
	HUVECs and MSCs	√				

ITS, Insulin-Transferrin-Selenium; FGF, fibroblast growth factor; BMP, bone morphogenetic protein; FCS, fetal calf serum; HGF, hepatocyte growth factor; KGF, keratinocyte growth factor; HCM, Hepatocyte culture medium; FBS, fetal bovine serum; NEAA, Non-Essential Amino Acids; HUVEC, human umbilical vein endothelial cell; MSC, mesenchymal stem cell; EGM, endothelial growth medium.

√ indicates that the culture medium contains the relevant components.

Recently, novel techniques have been incorporated into liver organoid generation. To manipulate gene expression, CRISPR/Cas 9, a powerful genome editing and engineering approach, has been applied for disease modeling and been utilized to fully understand the pathogenesis and pathophysiology of different kinds of diseases, among which the most popular one is cancer. Guan et al. discovered that phosphatidylethanolamine biosynthesis pathway was important to both early liver development and growth of HCC as well (43). For cholangiocarcinoma, it was found that loss of function of tumor suppressor gene BAP1 promoted malignant changes by disturbing chromatin accessibility (44). Other liver diseases investigated by means of CRISPR/Cas9 includes mitochondrial DNA depletion syndrome (40), hepatitis A virus infection (45), urea cycle disorders (46) etc.

## Recapitulation of parental tumor features

The most prominent advantage of PDOs is that, compared to conventional 2D models, 3D organoids restore parental tumor characteristics more faithfully, in terms of gene expression profiles, phenotypes, histological architecture and tumor subtypes (15, 47, 48). Mori and Kida revealed that culture dimensionality greatly influenced gene expression pattern, as evidenced by different expression level of genes involved in drug metabolism, such as CYP2D6, CYP2E1, NNMT and SLC28A1 (49). Different gene expression patterns result in different phenotypes. In 2D cultures, tumor cells displayed an epithelial phenotype and could only gained mesenchymal properties after cultured in 3D tumoroids. Histologically, HCC organoids could form pseudoglandular rosettes while iCCA organoids exhibit extensive glandular domains, with carcinoma cells invading the lumen and growing as cribriform structures (23). What’s more, organoids could also express diagnostic markers of corresponding PLC subtypes. HCC organoids but not iCCA organoids express high level of AFP while iCCA organoids express typical cholangiocarcinoma markers, such as CK7, CK19, EpCAM, nuclear of TP53 etc. (50).

**Figure 2:**
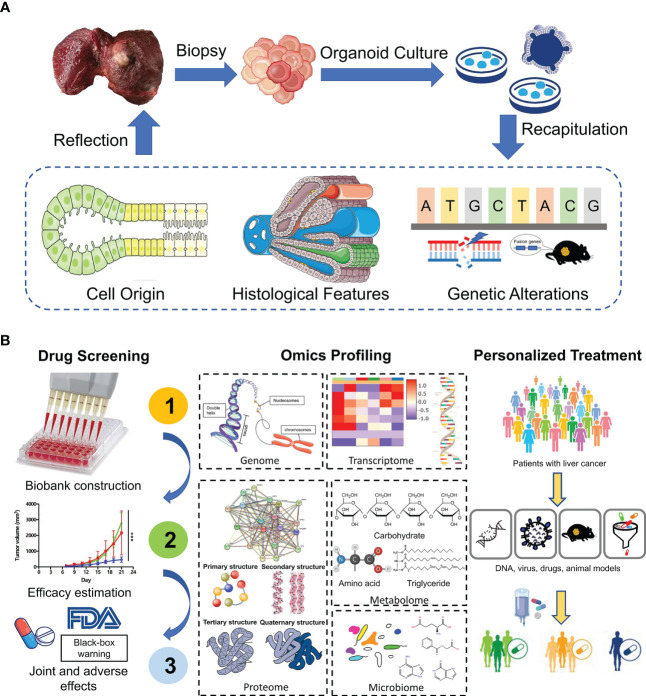
Application of liver organoids in precision medicine. **(A)** Patient-derived organoids from tumor biopsies reveal cell origin of PLC and recapitulate the histological and genetic features of original tumors. **(B)** PDOs show great potential in drug screening, omics profiling and personalized treatment.

Additionally, genomic and transcriptomic alterations vary from patients to patients even in different zones inside the same tumor (51). Therefore, molecular characteristics of PLC play a critical role in accurate diagnosis, biomarker discovery and development of new therapeutic approaches. By conducting genomic-wide transcriptomic analysis, Broutier et al. found that expression profiles of liver tumoroids resembled that of original tissues and subtypes. What’s more, organoids could retain the expression pattern in a patient-specific manner, e.g. genetic alterations. Research found that nearly 92% of global variants found in HCC biopsies were observed in the corresponding HCC organoids. And among these global variants, the preserved cancer-related somatic variants accounted for about 84% (23). Similarly, Yuan et al. adopted single-cell RNA sequencing to classify single cells derived from gallbladder carcinoma tissues and matched organoids into seven major distinct cell-type clusters. Interestingly, cells in each subtype were reclustered by their patients of origin (52). Taken together, the continuity of expression profiles and mutation landscapes from parental tumor tissues to organoids offers us an ideal insight into tumorigenesis and progression, facilitating clinical decision-making (**Figure 2**).

## Advances of PDOs in drug screening and personalized therapy

The available molecular strategies for advanced PLC are still limited until now (3, 53, 54). Systemic multi-kinase inhibitors, sorafenib (8) and lenvantinib (55), are the first line therapeutic drugs, while regorafenib (56), cabozantinib (57) and ramucirumab belong to second line therapy with low response rate. However, thanks to research progression, we seem to see the sign of hope for breakthroughs in immunotherapy. A phase III study demonstrated that compared with sorafenib alone, combination of atezolizumab (PD-L1 inhibitor) and bevacizumab (monoclonal antibody of VEGF) improved the median progression-free survival of unresectable HCC from 4.3 months to 6.8 months (58). Regardless, more targets are urgently needed to be found for treatment of advanced PLC. The property of liver tumoroids to finely recapitulate different aspects of the original tumors lays the foundation for its application in precision medicine, a significant one of which is large-scale drug screening and discovery of drug targets. Broutier et al. performed a proof-of-concept drug-sensitivity test and revealed that ERK inhibitor was effective in cells with BRAF- and MEK- inhibitor resistance (23). What’ more, correlation between drug sensitivities and mutational profiles in tumoroid lines was also identified. For instance, one tumoroid harboring mutations in CTNNB1 gene was resistant to porcupine inhibitor LGK974 (23). Besides, in an HTS of a total of 129 drugs in a large cohort of liver tumoroid lines, nine drugs belonging to five classes of antineoplastic agents were pan-effective, only two of which had ever been tested as systemic chemotherapy in liver cancer and one in cholangiocarcinoma (59). Except for drug screening by PDOs, we can also use PDOs to explore the mechanism of drug resistance. Wang et al. discovered that a hedgehog signaling inhibitor (GANT61) could potently suppress the growth of CD44+ sorafenib resistant HCC PDO lines, indicating combination of sorafenib and Hedgehog signaling inhibitor may benefit HCC patients with high CD44 expression (60). Furthermore, with metformin gradually accepted as a powerful anticancer agent, a recent study found that it could enhance the sensitivity of PDOs to methotrexate by decreasing the level of DHFR, a type of one-carbon metabolism enzyme (61). However, as therapy-resistance is a dynamic process with a series of genetic alterations and epigenetic regulation, key molecules responsible for reversing therapy resistance are not unchangeable. So exploring how these changes occur by PDOs will help us understand how cancer evolves and how drug resistance develops. Roerink et al. took tissues from different sites in a single primary tumor, isolated them into single cells and produced clonal organoids. Further, they used these organoids to monitor the shift of mutational landscape by phylogenetic trees along cancer progression and chemoresistance acquisition (62, 63).

In the early 21st century, the breakthrough in genome editing and RNA interference technology provided us a powerful tool in investigation of gene function (64). B. Artegiani et al. described that liver organoids with four common cholangiocarcinoma mutations (TP53, PTEN, SMAD4, and NF1) gained malignancy after loss-of-function of BAP1 by CRISPR/Cas9, because BAP1 could control the expression of junctional and cytoskeleton components by regulating chromatin accessibility (44). Li et al. generated paired organoids of primary tumors and matched liver metastases from the same CRC patients. Through gain-and-loss experiments, SOX2 was confirmed as a key regulator in CRC progression and liver metastases (65). Similarly, YAP1 and c-myc were also found to play important roles in CRC initiation and to be a potential therapeutic target (26, 66).

Another application of PDOs is to predict patient responses in clinical trials. By comparing responses of PDOs to anticancer agent with that of patients in clinical practice, we found that PDOs can recapitulate patient responses in clinical trials. S. Nuciforo et al. tested the efficacy of sorafenib on three iCCA organoids and found one of them derived from a rare subtype of iCCA (lymphoepithelioma-like CCC) responded, though sorafenib has not been subjected to the treatment of iCCA (24). The results were reproduced in a multicenter prospective study conducted by Luo et al. in 2017 (67). The prediction ability of PDOs has also been confirmed in other solid tumors including rectal cancer (68–71), breast carcinoma (72), and esophageal cancer (73).

## Reflection of PLC cell origin

In addition to application in drug therapy, PDOs also help us investigate deeper into origins and subtypes of PLC. HCC and iCCA are two main histological types of PLC with high plasticity and fate of transdifferentiation (74). Xue et al. demonstrated that combined type and mixed type cHCC-ICCs (combined HCC and iCCA) are two distinct molecular subtypes while the former one is more similar to iCCA and the latter one is more similar to HCC, indicating that HCC and iCCA may have monoclonal origins (75).

Earlier in this decade, Fan et al. and Sekiya et al. employed mouse models of iCCA and demonstrated that iCCA could be generated from transthyretin-positive hepatocytes through activation of the Notch and Akt signaling pathways (76, 77). This finding suggested a brand-new mechanism of iCCA origin and offered a promising target strategy for iCCA therapy. Saito et al. found that culture of iCCA organoids with differentiation medium significantly induced upregulation of hepatic markers, including albumin, CYP3A4 and HNF4A, increased the amount of bile acid production, but suppressed Wnt signaling pathway and the downstream protein DNMT1/3B, which indicates that iCCA could evolve towards hepatic direction after transdifferentiation induction (78). Conversely, Rimland discovered that Wnt promotion stimulated expression of ductal markers in intrahepatic bile duct organoids (18). These results imply that Wnt signaling pathway may play a key role in transdifferentiation between hepatic and biliary lineage. Besides, tissue-specific microenvironment have been shown to be able to promote transdifferentiation of iCCA organoids. Willemse et al. found tissue specific liver extracellular matrix could facilitate hepatic transdifferentiation with upregulation of hepatic markers, including albumin, CYP-3A4, HNF-4α, MRP-2. In turn, HCC could also gain cholangiocellular-like phenotypes during cancer progression (79). All in all, liver organoids offer us a reasonable model to explore PLC progression and evolution.

## Liver tumoroids in TME modeling and research

### Extracellular matrix as scaffolds for liver tumoroid modeling

Extracellular matrix (ECM) is one of the most critical acellular components in TME, which mainly includes collagen, elastin, glycosaminoglycans and other associated proteins and small molecules. ECM has tissue-specific physicochemical properties to accommodate to cell-to cell and cell-to-matrix interaction. These activities are closely related to cell behaviors, such as proliferation, differentiation, migration, angiogenesis etc. (80). Thus, faithful restoration of disease-specific ECM is important to generate models for disease research. Matrigel is now the most commonly used material to mimic ECM in 3D models. However, as Matrigel is extracted from Engelbreth-Holm-Swarm (EHS) murine sarcoma, there are many problems including batch-to-batch variation, ill-defined properties, possibility of transfer of pathogens etc. (81, 82) Therefore, there needs to be some modification or improvement of Matrigel.

Lee et al. manufactured a 96-pillar/well plate as a cell container with Matrigel to create an aggregated spheroid model for HCC (83). In this model, Matrigel was attached to the surface of a 96-pillar plate with HCC cells embedded in it. Cells assembled at the curved end of the Matrigel because of gravity to form a large single spheroid. Compared with many small diffuse spheroids generated by conventional methods, these aggregated spheroids were more suitable for drug screening and TME simulation because of the higher drug resistance, limited drug penetration ability and more marker expression. However, a high-quality organoid should not only recapitulate phenotypic and genomic features of original tumors, but also retain the biochemical and biophysical properties, which are crucial for cell activities and can affect gene expression in turn (84, 85). Hence, there emerges some other matrices as alternatives to Matrigel to mimic the tumor mechanical environment. Fong et al. fabricated a 3D cellulose-based sponge system with interconnected macropores which enabled spheroid size control through physical constraint (86). By culturing HCC patient-derived xenograft (PDX) cells in the hydrogel, this model sustained the mechanical properties and omics profiles as *in vivo* PDX models but failed to support metabolic activities as *in vivo* tissues. There may be one problem with the HCC-PDX derived model that cells from xenograft tissues contain host-derived ECM and stromal cells, leading to the possibility of deviation from original tumor tissues (87). To improve, Dong et al. mixed suspended alginate-gelatin hydrogel with patient-derived liver tumor multicellular clusters and acellular components, which guarantees to well recapitulate the landscape of original tumors (88). Besides, novel biomaterials, such as silk, are utilized in bioengineering due to its biocompatibility, supportive capacity and processing feasibility of fibroin. Silk-derived fibroin forms a bioactive composite scaffold, in which arginine-glycine-aspartate motifs provide domains for cell adhesion and organoid growth (89, 90). However, as tissue stiffness plays an important role in biological processes of liver cancer including tumorigenesis, angiogenesis, metastasis and epithelial to mesenchymal transition (91, 92), there are few 3D liver tumoroids to model this extracellular characteristic and it may be a novel research orientation in the future.

### Cellular components as crucial parts of TME

Except for ECM, different kinds of cells participate in the formation of TME, including endothelial cells, immune cells, fibroblasts, stromal cells etc. These cells are closely related to tumorigenesis, progression, metastasis and patients’ prognosis (93, 94).Therefore, simulation of the composition and behavior of cells in TME is critical to portray the whole landscape of original tumors.

Angiogenesis and vascular remodeling greatly contribute to the progression of HCC (95, 96). To model this pathologic process, endothelial cells were co-cultured with HCC cells to generate vascularized organoids (34, 97). A recent article investigated the angiocrine crosstalk in ex vivo organoids and revealed that activation of tumor necrosis factor (TNF) signaling and polarization of macrophages in the TME were induced by endothelial cells, indicating the possible interaction among these cells (97). Immunotherapy, depending on enhanced self-immune activities, is now emerging as a powerful tool in anti-tumor strategies. To overcome the problem that primary T cells are sensitive to unfavorable external stimuli, Zhang et al. coated the surface of T cells with a flexible DNA network as a protective scaffold. Tumor-infiltrating cells (TILs) modified *via* this coating method showed potent tumor killing activities as evidenced by increased antitumoral cytokines and apoptotic cells (98). Besides, chimeric antigen receptor T (CAR-T) cell therapy is another promising cancer immunotherapy. Zou et al. generated HBV surface protein-specific CAR-T and personalized tumor-reactive CD8+ T cells (99). When co-cultured in autologous HBVs+ HCC organoid, these T cells exhibited stronger anti-tumor behaviors than normal and might provide a therapeutic option in the future clinics.

Cancer-associated fibroblasts (CAFs) are a group of heterogenous cells in tumor stroma (100). Currently, CAFs are widely accepted as liver tumor progression promoters mainly by two ways; one is direct effects on tumor cells *via* paracrine signaling, exosome transfer and physical contact, and the other is indirect pattern by communication with stromal cells (101). Building *in vitro* TME by culturing CAFs in liver tumoroids not only confirmed the effects of CAFs on tumor growth and drug resistance, but also revealed the feedback regulation of CAF phenotypes by paracrine signals from HCC cells (102).

**Figure 3:**
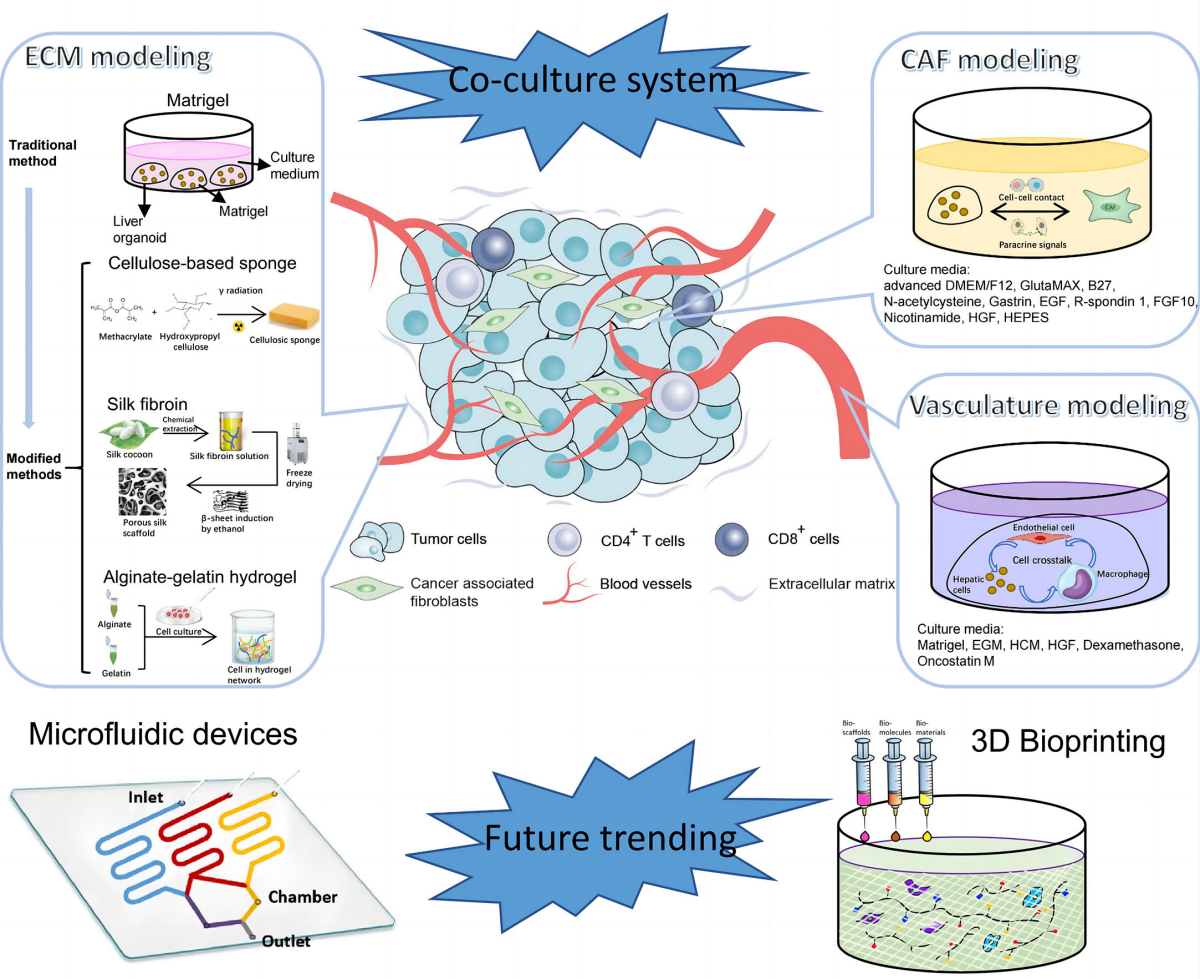
Establishment of tumor microenvironment (TME) of PLC. TME of PLC is made up of complex components, including extracellular matrix, cellular components like vascular endothelial cells, immune cells, CAFs, and etc. The diagram illustrates components in TME of PLC and steps for *in vitro* model construction. CAF, cancer associated fibroblast; EGM, Endothelial Cell Growth Medium; HCM, Hepatocyte culture medium; HGF, hepatocyte growth factor; EGF, epidermal growth factor; FGF, fibroblast growth factor.

Some research cultured nearly all the typical types of non-parenchymal cells in organoids to mimic TME in a more comprehensive way. An HCC organoid with cancer cells, fibroblasts, endothelial cells and ECM inside expressed more markers related to angiogenesis, inflammation and EMT compared with organoids containing tumor cells alone, emphasizing the significance of non-parenchymal components in the formation of 3D organoids (103). What’s more, Qiu et al. introduced iPSC-derived endothelial cells and mesenchymal cells into HCC organoids and orthotopically implanted into the liver of immune-deficient mice with various diseases. Experiments demonstrated that fibrosis TME could promote tumor amplification while non-alcoholic hepatic diseases couldn’t (104). This implies that specific environment may contribute to the progression of liver cancer. Although different, all these techniques try to apply tumor cells and non-parenchymal cells in one co-culture system to faithfully mimic *in vivo* TME and explore the interaction among all the components in it (**Figure 3**).

### Other novel techniques for TME modeling in the promising future

Except for coculture, there springs up other novel technologies to equip organoids with more advanced functions and make models more comparable to naïve tissues in terms of vasculature, spatial arrangement and other details. Some studies reported that pluripotent stem cells cultured in Matrigel would finally differentiate into liver organoids with various characteristics due to the nonuniform cell microenvironment and intercellular communication (42, 105). To improve, micropatterning technique was employed to determine the location, arrangement and size of each organoid to ensure the homogeneity of all cultured organoids, and avoid the unreliable results generated from heterogenous organoids (105). In addition, microfluidic devices provide a platform to mimic the circulation system *in vitro*. By incorporating multiple microscale channels with different structures, this tool allows researchers to manipulate the dynamic fluid flow and gradients of gas and soluble molecules to better mimic the biochemical and biophysical processes (106). For example, a microfluidic vascular bed could be constructed by incorporating microfluidic chips underneath a 384-well microtiter plate to allow for the formation of a stable vascular network in the targeted liver tissue (107). There are many other methods to engineer the vasculature in the organoids (108), which is promising to be utilized in liver tumoroid establishment in the near future. In addition, cells are able to experience shear stress and hydrostatic pressure in microfluidic devices, which may change immune cell function in the TME and thus the interaction with tumor cells (109, 110). Through combination of organoids and microfluidic device, Rajan et al. successfully fabricated an organ-on-a-chip platform with multiple humanized organ constructs. This device helps to study the *in vivo* drug metabolism, which is mainly accomplished by liver, and the possible drug toxicity to other tissue organs (111). Apart from drug research, immune activities can also be screened. Natarajan et al. generated a liver organoid system using a microfluidic chip, in which coculture with HCV specific CD8+ T cell assists in monitoring and understanding the adaptive immune responses to HCV invasion (112). 3D bioprinting is another potential powerful technology for tumor modeling. By fabricating 3D biomimetic organ-like or tissue structures with physiological microarchitecture and microenvironment in a computer-controlled way, this technique possesses numerous advantages, such as simulation of physical parameters of ECM including stiffness and ultrastructure, and construction of perusable vascular networks (113, 114). Bioprinting has a wide range of applications, different types of which have their distinct usages. For example, Bernal et al. used volumetric bioprinting to construct liver organoids to study the metabolic function of liver (115) and Maloney et al. applied immersion bioprinting to generate homogenous cancer organoids in 96-well plates for high throughput of 3D drug screening (113). Despite the fact that there are few researches concerning these novel techniques, based on the existing studies, it’s still prospective that utilization of the new tools would shed light on liver cancer biology and personalized treatment for different patients.

## Current limitations

Although liver organoids outperform current models to a great extent and the prospect for applications of PDOs in the future liver cancer research looks bright, there are still some limitations which remain to be solved for clinical translation.

### High costs but low success rate of liver tumoroid establishment

Currently, Matrigel is the most commonly used ECM in liver tumoroid culture and different kinds of growth factors are important for specific differentiation and induction. These two kinds of materials are all costly, which may hinder the scalable production of liver PDOs. Besides, the culture process can be time consuming as well. According to the experience of Broutier et al., the average duration of first passage for liver tumoroids is 2-3 weeks, which is about 1 week longer compared with that of healthy liver-derived organoids (23). And it takes as long as 4-12 weeks in order to obtain sufficient models for drug screening (23). Patients may not be able to wait such a long time to get the appropriate treatment strategies, especially in cases of emergency. Despite the high costs of money and time, success rate of liver tumoroid culture is relatively low in comparison to other tumor organoids and varies greatly between hepatocyte and cholangiocyte origin. For example, success rates of organoid establishment are reported to be around 90% for colorectal cancer and gynecologic tumors (116, 117). However, for establishment of HCC organoids derived from needle biopsies, success rate is only approximately 30% (24) while 50% for that of iCCA organoids (118). Various reasons could account for this. First, compared with cancer tissues, normal tissues have more robust proliferation ability to form organoids in the early stage of construction due to the gradual telomere shortening (23, 118). Therefore, normal tissues within the tumor samples would be the source of contamination and further outcompete the tumoroids. Furthermore, liver tumoroids could only be successfully generated from poorly differentiated tissues with >5% proliferating cells (23). In addition, the frequency of driver gene mutations such as TP53 and KRAS in biliary tract cancer is relatively low, which causes lack of promotion for tumoroid amplification (119). To overcome the above issues, sample selection and purification, proper genome editing, addition of growth factors are several methods to improve organoid yields.

### Irreproducible and heterogenous nature of current liver tumoroids

Although it’s widely accepted that organoids could finely recapitulate the original tissues in terms of genetic profiles, histological architecture etc., some studies also reported that there existed transcriptomic differences between primary tumors and the corresponding tumoroids (34, 36, 118). This may impair organoids’ ability to precisely reflect the nature of parental tumors and restrict the implementation of clinical treatment. This genetic disparity is partially limited by the current uncontrolled and non-standardized protocols for organoid culture (48). For example, tissues from different regions of biopsy samples contain different kinds and proportions of cellular and noncellular components, leading to heterogenous organoids despite origin of the same specimen (118). Furthermore, there is still no consensus on the procedure of liver organoids generation, including steps and processes, culture medium, etc. Researchers have made large amounts of modifications but whether they can be replicated has not been validated yet. Although investigators have explored to integrate various cellular components in TME into liver tumoroids, as mentioned above, most of the current models consist of only liver cells and noncellular matrix. Lack of stromal cells will distort tumor cell behaviors, causing unfaithful representation of original tissues. Besides, Matrigel is exogenous matrix extracted from murine sarcoma and there exists certain discrepancy from ECM of specific patient. Hence, to facilitate scalable production of liver tumoroids of high quality, unified protocols are needed and modern techniques are in progress to construct complicated and precise organoids, such as coculture systems and microfluidic devices (120).

## Conclusion and perspectives

Currently, PDO is a functional 3D model for basic and translational research of various types of cancer. Due to the faithful recapitulation of molecular profiles and histological architecture of parental tissue, liver tumoroids have helped researchers to further comprehend the complexity of PLC, in terms of tumor initiation, progression, metastasis, drug screening and personalized treatment. Although great advances have been achieved, PLC is still a mixed type of malignant tumor with relatively poor survival. Therefore, new targets and treatment strategies are urgently needed and PDO is such a practical tool for deeper exploration.

Dating back to 2015, Clevers et al. established the first living cancer organoid biobanks containing a set of 20 genetically diverse CRC specimens and matched healthy organoids (117). Since then, organoid biobanks of pancreas (121), bladder (122), breast (72), prostate (123) etc. have been established in succession. Such specimens of the living biobanks are characterized with multi-omics data and detailed clinical annotation, providing us an ideal platform for molecule subtype classification and application of precision medicine. If liver organoid biobank is constructed in the near future, it will greatly accelerate the progression of liver disease research. In conclusion, despite the current existing limitations, liver organoids will be an indispensable tool in future research.

## Author contributions

YC and YL contributed to the manuscript’s composition, literature review, and drafting and finalization of the manuscript. LZ and SC contributed to the literature review and search. XL contributed to the manuscript’s drafting and critical review. YM contributed to the approval of the final version of the manuscript. All authors contributed to the article and approved the submitted version.
